# Isolation and Characterization Through Whole-Genome Sequencing of STEC Strains from Free-Ranging Red Deer

**DOI:** 10.3390/vetsci12100929

**Published:** 2025-09-24

**Authors:** Stefania Lauzi, Rosangela Tozzoli, Paola Chiani, Matteo Nava, Arnold Knijn, Valeria Michelacci, Stefano Giacomelli, Gaia Scavia, Stefano Morabito, Camilla Luzzago

**Affiliations:** 1 Department Veterinary Medicine and Animal Sciences, Università degli Studi di Milano, 26900 Lodi, Italy; matteo.nava@unimi.it (M.N.); camilla.luzzago@unimi.it (C.L.); 2 Department of Food Safety, Nutrition and Veterinary Public Health, Istituto Superiore di Sanità, 00161 Rome, Italy; rosangela.tozzoli@iss.it (R.T.); paola.chiani@iss.it (P.C.); arnold.knijn@iss.it (A.K.); valeria.michelacci@iss.it (V.M.); gaia.scavia@iss.it (G.S.); stefano.morabito@iss.it (S.M.); 3 ATS della Montagna, 23100 Sondrio, Italy

**Keywords:** STEC, wild ruminants, red deer, *Escherichia coli* cross-pathotypes

## Abstract

Whole-genome sequence (WGS) analysis was used in this study to characterize Shiga toxin-producing *Escherichia coli* (STEC) isolates in free-ranging red deer from the central Italian Alps. Fecal samples from 92 hunted red deer collected between September and December 2022 were analyzed for the presence of STEC. Single *E. coli* colonies positive by PCR for *stx* genes were analyzed by WGS. STEC were isolated from eleven (12%) samples, showing eight *stx2b*, one *stx2a*, two *stx1c*, and one *stx1a* subtypes. Different serotypes and sequence types were identified (*n* = 8 each). Three isolates of O27:H30 serotype and ST753 showed no correlation in the cgMLST analysis (AD range 44–98). All strains harbored additional virulence factors. The only isolate harboring *stx2a* also possessed the *eae* gene and belonged to serotype O26:H11. Some isolates displayed shuffled virulence features of more than one *E. coli* pathotype. The high genetic diversity of strains circulating in the red deer population living in the central Italian Alps, including the STEC O26:H11 strain associated with STEC from severe disease in humans, confirms red deer as STEC reservoirs and highlights the need for monitoring the presence of these pathogens in wild ruminants.

## Simple Summary

Shiga toxin-producing *Escherichia coli* (STEC) are important human food-borne pathogens and red deer have been recently identified as STEC carriers. This study aimed to isolate and characterize by whole-genome sequencing (WGS) STEC strains from free-ranging red deer in the central Italian Alps. Fecal samples from 92red deer hunted between September and December 2022 were analyzed by bacteriology and WGS analysis. STEC strains were isolated from 11 (12%) samples. Different genetic features were identified in STEC strains, which included one isolate belonging to serotype O26:H11 and possessing the *stx2a* subtype along with the *eae* gene, frequently associated with severe disease in humans. Our results show a wide range of strain types, including some with cross-pathotype features, present in a red deer population from the central Italian Alps. Results underscore red deer as STEC carriers and confirm the need of STEC monitoring in wild ruminants to understand the potential role for these animals in the transmission of infections to humans.

## 1. Introduction

Shiga toxin-producing *Escherichia coli* (STEC) are important zoonotic food-borne pathogens. A total of 10,217 cases of STEC infection in humans were reported in 2023 in countries from the European Union and European Economic Area [1]. STEC-induced disease in humans is characterized by a variety of clinical conditions ranging from mild intestinal disorders to hemolytic uremic syndrome (HUS) and kidney failure, which are life-threatening conditions [2].

The most frequently detected STEC strains in HUS patients belong to serogroups O26, O103, O111, O145, and O157, and have been termed as top-five STEC serogroups [3].

However, STEC serogroups, while regarded as useful for epidemiological investigations, do not reflect the pathogen potential of the isolates [4]. Indeed, the recent pathogenicity assessment of STEC exercise carried out by the European Food Safety Authority (EFSA) indicates that humans’ most severe clinical pictures are not correlated with specific STEC serotypes. The pathogenicity of STEC is rather associated with certain Shiga-toxin subtypes and specific colonization factors enabling attachment to the intestinal epithelial cells [4].

Consumption of contaminated food products of animal origin has been identified as an important transmission route of STEC strains to humans [5]. However, other routes of transmission of STEC infection are increasingly reported, including contaminated water, person-to-person spread, contact with animals or animal feces, and visiting farm environments [6,7]. The natural reservoir of STEC is known to be ruminants, especially cattle, which shed the microorganism with feces [8]. More recently, wild ungulates have been investigated as possible STEC reservoirs. In this respect, deer species from different European countries, the United States, and Japan have been reported to harbor STEC strains in feces [3,9,10,11,12]. Moreover, red deer may harbor STEC strains for prolonged periods, mirroring the long-term shedding of STEC in cattle [13]. STEC strains have been detected in carcasses of wild ruminants, including hunted animals [14]. The popularity of wild game meat consumption has increased in recent years [15] and STEC strains from wild ruminants have been reported to cause severe diseases in humans, although rarely [2,16,17].

The importance of STEC monitoring in wildlife has been highlighted according to the One Health perspective [18]. This is particularly important in the case of wild populations of ungulates, such as red deer (*Cervus elaphus*), which have been increasing their densities and distribution across Europe in recent decades [10]. This could generate interfaces with humans and domestic animals that may eventually be routes for the transmission of pathogens [19].

Free-ranging red deer in Stelvio National Park (SNP) have been reported as carriers for LEE-negative, *subAB*-positive STEC, possessing features overlapping those of STEC strains causing human disease [12]. The aim of the present study is to follow up and expand the previous investigation [12] and to extend the findings to other areas bordering the large area of the SNP. The presence and the characterization of STEC strains isolated from red deer living in their natural habitats living at the edges of this national park have been determined to obtain deeper insights into STEC circulation in this wildlife setting.

## 2. Materials and Methods

### 2.1. Study Area and Deer Sampling

The study area is located in Valle Camonica, an alpine area of the Lombardy region, northern Italy. Specifically, the study area is within the Alpine Hunting District of Ponte di Legno (CAC1), with elevations ranging from 1000 to 2700 m above sea level. The environment is typically alpine, characterized by coniferous forests at lower elevations and alpine meadows at higher altitudes. Among wild ungulates, the red deer is the most abundant. The study area was the hunting-free zone within the CAC1 of about 9500 hectares located outside the SNP and the Adamello Regional Park. The area hosts an estimated deer population of 1200–1300 individuals with a density of 12 deer/100 hectares, which is annually counted by spring spotlight [20].

The red deer investigated in this study for the presence of STEC were legally hunted. Therefore, animals were not sacrificed for research purposes specific to this study. The individuals included in this study were all wild and free-living and were hunted between September and December 2022. After hunting, inspection under the supervision of a veterinarian was performed on all animals, which were immediately brought to the control center of CAC1. Data on hunting site, month of sampling, sex, and age were obtained for each individual. Animals were divided into three age categories: calves (<1 year old), yearlings (1 year old), and adults (≥2 years old). Fecal samples were collected directly from the rectum and were stored at −20 °C immediately after collection. Frozen fecal samples were brought to the laboratory monthly for further analysis.

### 2.2. Bacteriological Analysis and STEC Detection

Bacteriological analysis was performed according to the adaptation of ISO/TS 13136:2012 [21], with minor modifications. A total of 1 g of each fecal sample was enriched into 9 mL of buffered peptone water (BPW; Microbiol Diagnostici, Cagliari, Italy) in aseptic conditions. The sample was incubated overnight at 37 °C. One mL of the BPW enrichment culture was centrifuged at 11,000× *g* for 5 min and the pellet was resuspended in one mL of sterile distilled water. DNA was extracted by boiling 50 µL of the resuspended pellet. Extracted DNAs were tested for the presence of *stx* genes according to previously published qPCR protocols for *stx1* and *stx2* genes [22]. A PCR reaction was performed for the identification of the pools producing *stx2f* gene subtype, which is not detected by the qPCR protocol used, as described elsewhere [23]. In the presence of negative qPCR results, the sample was considered negative.

BPW enrichment cultures with qPCR-positive results were subsequently plated on MacConkey agar and incubated at 37 °C for 18–24 h. Following incubation, 50 single typical *E. coli* colonies were subsequently selected from the MacConkey agar plate for each sample, point inoculated on nutrient agar as a back-up, and DNA was extracted using the boiling method from each of the 50 selected colonies. DNAs from the 50 colonies of single animals were pooled. Each pool was composed of 10 colonies each and tested by conventional PCR [24]. A fecal sample with a negative *stx* PCR result in the 50 tested *E. coli* colonies analyzed in pools was considered as presumptive positive [21]. In the presence of *stx*-positive pooled samples, further analysis of DNA from single colonies composing the pools was performed using the same PCR protocol in order to confirm the presence of strains possessing *stx1* and/or *stx2* genes.

Confirmation of *E. coli* species identification of the strains possessing *stx1* and/or *stx2* genes was obtained by the MBT Microflex LT/SH matrix-assisted laser desorption/ionization–time of flight mass spectrometry (MALDI-TOF MS) (Bruker Daltonik GmbH, Bremen, Germany), following previously described methods [25]; these were designated as STEC. A fecal sample with ≥one isolated STEC colony was considered STEC-positive. The mean and range of the quantification cycle (Cq) values were recorded for positive and presumptive positive samples. The presence of the *eae* gene was tested for the isolated STEC strains by conventional PCR [24]. Single STEC colonies/animals were selected based on the *stx* profile and were further subjected to whole-genome characterization.

### 2.3. Whole-Genome Sequencing and Strains Characterization

Eleven STEC strains were subjected to whole-genome sequencing (WGS) and analysis. STEC strains were cultivated in Trypticase soy broth (TSB) and incubated at 37 °C overnight for 18–24 h and total DNA was extracted from 2 mL of these overnight cultures using the E.Z.N.A. Bacterial DNA kit (Omega Bio-tek, Norcross, GA, USA). Extracted DNA was sequenced using the Ion Chef and Ion Torrent S5 instruments (Thermo Fisher Scientific, Waltham, MA, USA), with the template preparation and sequencing run performed with Ion 510™ & Ion 520™ & Ion 530 kit (Thermo Fisher Scientific, MA, USA) following the manufacturer’s instructions for 400 bp DNA libraries (NEBNext^®^ Fast DNA Fragmentation & Library Prep Set for Ion Torrent, New England Biolabs, Ipswich, MA, USA). Only for one strain (ED1684) was the sequencing outsourced to Eurofins Genomics (Ebersberg, Germany), where a 150 bp sequencing protocol was applied on a NovaSeq Illumina platform (Illumina, Inc., San Diego, CA, USA).

The analysis of the sequences has been conducted through the IRIDA-ARIES genomic surveillance information system, deployed and maintained at the Istituto Superiore di Sanità [26]. The PHANtAsTiC 2.1 pipeline (https://github.com/aknijn/phantastic-galaxy, accessed on 23 May 2024) was used to perform all the bioinformatic analyses [26]. This pipeline is designed to work on sequencing data generated on either IonTorrent or Illumina platforms [26]. During the typing phase, this workflow analyses and provides results on the serotyping, Multilocus sequence typing (MLST) and antimicrobial resistance (AMR) prediction by assembling the reads in contigs and performing BLASTn with specific tools and databases, as previously described [26], as well as virulotyping through reads mapping against *E. coli* virulence genes database [26]. Aside the molecular typing, a cluster analysis is performed on the distance matrix of the core genome cgMLST profile of each sample with respect to those of all samples of the same serogroup present in the platform, providing indication on the phylogenetic relatedness among strains.

The PHANtAsTiC pipeline could not assign the *stx* subtype of two strains (ED1683 and ED1685). For these two strains, the Shiga toxin typer tool v2.0 (https://github.com/aknijn/shigatoxin-galaxy, accessed on 31 July 2025) was used for *stx*-subtyping. This tool is an optimized BLASTn search against the sequences database of *stx* subtypes developed by the Statens Serum Institut (https://bitbucket.org/genomicepidemiology/virulencefinder_db/src/master/stx.fsa, accessed on 31 July 2025) through the public ARIES Galaxy server [27], and provides detailed results on the percentage of sequence identity for the best match in the database.

The whole-genome sequences (WGS) of the 11 STEC strains have been uploaded to the European Nucleotide Archive (ENA) public repository (Project Accession no. PRJEB95802).

### 2.4. Statistical Analysis

The association of STEC presence with sex, age, and month of collection of hunted red deer was evaluated using a Pearson’s chi-square test or Fisher’s exact test. Epitools (https://epitools.ausvet.com.au/, accessed on 31 July 2025) was used for statistical comparisons, using *p* < 0.05 as threshold for statistical significance.

## 3. Results

### 3.1. Detection of STEC in Animals

Fecal samples were collected from a total of 92 hunted red deer. Table 1 shows the characteristics of the sampled animals.

A total of 24 samples out of the 92 (26%) were negative in the real-time PCR assay aimed at detecting the *stx* genes. As for the 68 (74%) BPW-enriched cultures with qPCR-positive results, 57/92 (62%) were categorized as presumptive positive (real-time PCR assay positive for the presence of *stx*, but isolation of STEC strain out of 50 tested *E. coli* colonies not achieved). A total of 11 out of the 92 (12%; 95% CI: 5.3–18.6%) were positive samples (i.e., presence of STEC confirmed by the isolation of the microorganism), accounting for a recovery rate of 11/68 (16.2%). Based on qPCR results of BPW-enriched cultures, presumptive positive samples showed 31.6 Cq mean value (range 20–39.2 Cq) compared to 19.5 Cq mean value (range 18.5–21.8 Cq) observed in positive samples.

Out of the 50 *E. coli* colonies tested, the number of confirmed STEC colonies obtained from 1 positive sample ranged from 1 to 10. All the STEC colonies comprising the pools from the same animal showed the same *stx* profile and 1 STEC colony per animal was selected for all the 11 STEC-positive fecal samples.

Out of the 11 STEC isolates, *stx2* genes were found in 9 (81.8%) strains, whereas 3 (27.3%) isolates showed the presence of *stx-1* genes. The intimin coding gene (*eae*) was detected in one (9.1%) isolate. The *stx-2f* gene was never detected by PCR or WGS.

### 3.2. Molecular Serotyping, Multi-Locus Sequence Typing, Virulence Genes Assets

The short sequencing reads were assembled in contigs with a median N50 of 72,790 bp. The results of the WGS-based characterization of the 11 STEC isolates in this study are reported in Table 2. *Stx*-coding genes subtyping showed the detection of four different *stx* subtypes combinations among the isolates, with *stx2b* only being the most frequent, observed in seven (63.6%) isolates. For ED1683 and ED1685, for which the *stx2b* could be called after deeper inspection, 99.838% and 99.434% identity with the reference *stx2b* sequence was observed, respectively. One strain possessed the *stx2a* subtype and was of O26:H11 serotype. The complete range of *stx* subtypes detected in the red deer STEC strains is depicted in Table 2.

The 11 STEC isolates showed nine different serotypes and nine sequence types (Table 2). For one isolate, the O-group could not be determined (ONT). Isolates with the same serotype belonged to the same sequence type. Three strains belonged to the same serotype O27:H30 but the cgMLST analysis showed no correlation among them (allelic distances, AD, ranging from 44 to 98). Some of the STEC serotypes had been detected in STEC strains isolated in another study in the national park in 2018–2019, namely O146:H28, O27:H30, O91:H14, and O104:H7 [12]. Also in this case, the cgMLST analysis showed no correlation with the previously isolated STEC strains of the same serotypes (73–91 AD for the O146:H28, 44–98 AD for the O27:H30, 21–33 AD for the O91:H14, and 32 AD for the O104:H7).

One isolate belonged to serotype O26:H11, possessed the *stx2a* gene and the intimin-coding gene, *eae*, located in the LEE pathogenicity island and involved in the attaching and effacing lesion. This strain also presented the *efa1* gene, described previously to have an involvement in the colonization of certain LEE-positive STEC [28,29], as well as several genes associated with STEC strains possessing the LEE locus (e.g., *tccP*, *tir*, among others). All the other isolates were negative for the presence of the *eae* gene, in accordance with our previous observations [12] and as described in other studies [30].

As for the additional virulence genes patterns, the *aaiC* gene described in some Enteroaggregative *E. coli* (EAEC) strains [31] was detected in one O104:H7 strain harboring *stx1* gene (Table 2). However, the genes typically associated with EAEC such as the *aggR* and the *aat* genes were not detected in any of the isolates investigated.

Several strains showed the presence of putative STEC-associated colonization factors, including those encoded by the genes *iha*, *lpfA*, *saa* [32], and the *tia* gene [33]. The *iha* adhesion gene and the *tia* gene were the most prevalent and were found in eight strains, followed by the *lpfA* gene detected in seven strains. The *papC* gene typically associated with *E. coli* causing urinary tract infections was detected in one strain [34].

Five isolates harbored the *ehxA* gene, encoding the enterohemolysin toxin, which is considered as the hallmark of a large virulence plasmid frequently found in STEC strains [35].

The *subAB* gene was frequently detected, being present in 9 out of the 11 STEC strains investigated here, whereas the *astA* toxin gene was identified in 5 isolates. All the 11 STEC isolates possessed *terC* (involved in tellurite resistance), *gad* associated with acid tolerance, and the *traT* gene (transfer protein), responsible for inhibition of the classical pathway of complement activity and involved in the serum resistance [36]. Further virulence genes detected in the STEC strains from red deer included the *ireA* gene, encoding an iron-regulated outer membrane protein [37], and the *espI* gene coding for a non-LEE effector protein described in patients suffering from severe STEC-induced disease [38]. Both these genes were detected in 9 out of 11 strains. The gene *cba* coding for Colicin B produced by *E. coli*, and the *iss* gene associated with the serum resistance, as well as genes involved in the production of bacteriocins (*mchF*, *mchC*, *mchB*), were detected in eight strains each.

Interestingly, besides *papC*, the O54:H45 strain ED1667 presented several virulence genes described in pathogenic *E. coli* and other enteropathogenic bacteria, such as *senB*, encoding an enterotoxin described in uropathogenic *E. coli* (UPEC), and enteroinvasive *E. coli* (EIEC) [39]. This strain also possessed the *pic* gene, present in EAEC, UPEC, and *Shigella flexneri* strains [40]; *sitA*, involved in the iron metabolism in Salmonella Typhimurium [41]; and *vat*, coding for a vacuolating autotransporter toxin (Vat), a member of serine protease autotransporter proteins of *Enterobacteriaceae* (SPATEs) present in some UPEC strains [42]. Another strain, ED1678, displayed virulence genes associated with *E. coli* strains from urinary tract infections, such as *kpsMII*, described in *E. coli* strains from cases of cystitis [36] (Table 2).

### 3.3. Comparison of STEC Strains Sequences from Red Deer and Human from the Same Region

The IRIDA-ARIES platform collects WGS STEC data from isolates of both human and non-human origin, allowing the prompt comparison among circulating STEC strains in a specific region and at national level [26].

An analysis of human STEC strains sequences included in IRIDA-ARIES in the Lombardy region (about 300 isolates obtained in the period 1989–2025) showed the presence of STEC strains with similar characteristics of those isolated in red deer during this study. In particular, 62 several WGS data from O26 isolates are present in the database, most of which harbor *stx2a*. The data retrieved from IRIDA show that STEC strains belonging to serogroups O91 (*n* = 2) and O146 (*n* = 2) were also detected in humans in the Lombardy region. No cgMLST clusters have been identified so far in the STEC strain sequences from red deer and humans included in the IRIDA-ARIES platform [26].

### 3.4. STEC Distribution and Risk Factors for STEC Colonization

STEC presence according to sex, age, or months of sampling (Table 1) did not show significant differences among categories, with results showing *p* values of 0.5223, 0.2159, and 0.7165, respectively. The distribution of the 11 serotypes and sequence types according to sex, age, and month of collection are reported in Table 3. With respect to the shooting sites, the positive samples were distributed within three different municipalities (Ponte di Legno, Vione, and Vezza d’Oglio) and due to the large movements of the species, it was impossible to make any assumption concerning association of STEC positivity with the hunting sites.

## 4. Discussion

STEC are important food-borne pathogens, and it has been well documented that the gastrointestinal tracts of ruminants are the main natural reservoir of STEC [8]. Indeed, the role of farmed ruminants is well recognized in the epidemiology of STEC disease in humans [8]. However, STEC strains are ubiquitous in various ecosystem niches, and they should be addressed under the One Health approach [18,43]. In this perspective, wildlife, including wild ruminants, have been recently recognized as potential important reservoirs of food-borne pathogens, including STEC [5,11]. Wild ruminants can asymptomatically carry STEC strains capable of posing a risk to human health.

The present study showed fecal carriage of STEC strains in wild populations of red deer from the Italian Central Alps, causing the shed of these pathogens in the environment, in agreement with prevalence previously observed in cattle [2]. The presence of presumptive positive samples was not surprising as they are part of the flow diagram for STEC diagnosis [21]. Presumptive positive samples indicate the potential presence of STEC in the enrichment broth but further confirmation by isolating STEC colonies is required to identify a positive sample [21]. It has to be noted that, besides the screening step addressed by real-time PCR, consisting of a very sensitive assay, for the detection of STEC virulence genes, the confirmation step represents the bottleneck of the procedure, since no differential or selective media specific for STEC are available. In samples harboring low amounts of STEC or stressed bacteria, as well as containing high background microflora, the isolation of STEC may be very difficult or not achieved. Moreover, presumptive positive results may be due to the fact that *stx* virulence markers are located on a mobile genetic element, a bacteriophage, which may be lost, or may be harbored in free bacteriophages present in the enrichment broth [43]. Lastly, a positive qPCR result may not guarantee that the bacteria are viable or able to grow on a plate. The high percentage of presumptive positive samples, also reported in previous studies [18], may, in the present study, be attributed to the one-month storage of fecal samples at −20 °C, which could have reduced the viability of the STEC strains. Additionally, lower STEC loads in these samples, as suggested by the higher mean Cq values of the presumptive positive samples compared to positive ones, may have made it difficult to detect STEC strains among the colonies tested per sample. Unfortunately, the application of an enrichment step foreseen in the ISO/TS 13136:2012 diagnostic flow does not allow for defining the STEC load in fecal samples by qPCR analysis. On the other hand, the lack of enrichment may lead to underestimating the presence of this pathogen in fecal samples, when low amounts would be present. Future studies on the set up and application of quantification strategies of STEC in presumptive positive samples may provide indications on the impact of low bacterial load in the detection by ISO/TS 13136:2012. Alternative approaches that may help improve recovery rates and reduce potential underestimation of STEC prevalence include the prompt analysis of freshly collected samples, modifications of the ISO/TS 13136:2012 protocol increasing the number of *E. coli* colonies to analyze per sample, and the use of different selective media for STEC isolation [44]. Moreover, as a future perspective, the use of direct metagenomic analysis may help in the detection of STEC-indifferent specimens for the presence of STEC.

Despite the high presence of presumptive positive results, the STEC positivity observed in this study is in accordance with previous investigations on red deer [5] and is in agreement with the 19.9% prevalence already detected in red deer living in the bordering area of the Stelvio National Park [12]. Our results showing the presence of STEC in the population of wild ruminants of this study confirm the potential role of red deer as STEC carriers, highlighting the importance of wildlife in STEC shedding dynamics [13,45].

The role of STEC carriers is further confirmed by the detection of a wide range of STEC serotypes, comparable to those commonly associated with red deer, such as serogroup O146 [16,46] and similar to the ones previously detected in red deer from the bordering area of the Stelvio National Park (e.g., serotypes O146:H28, O91:H14, O27:H30, O104:H7). In addition, other STEC serotypes were detected in the animals ranging in the area concerned by this study, including O21:H21 (ST56), O54:H5 (ST491), O117:H4 (ST ND), and the LEE-positive O26:H11 (ST29), the latter being one of the six most common STEC serogroups in the EU in 2007–2022 [47].

Overall, our findings show that in this study, red deer predominantly carried LEE-negative STEC strains. Indeed, the absence of the *eae* gene in the genome has been suggested as an apparent feature of STEC typically shed by red deer [2,10,48]. Based on published risk assessment exercises [1,2], all these STEC strains isolated in this study may cause gastrointestinal disease and pose a threat for human health. Indeed, the STEC strains from this study, besides *stx* genes, also encoded adhesins, toxins, and other genes’ products already described in STEC isolated from disease in humans, such as the ones involved in the colonization of the host gut [32,33].

Several virulence genes may be harbored by mobile genetic elements, such as pathogenicity islands, plasmids, and bacteriophages, which can be transferred among bacteria. *Escherichia coli* is characterized by a huge genomic plasticity, and acquisition of different virulence genes by horizontal gene transfer plays a role in the emergence of specific *E. coli* pathogroups as well as pathogenic clones displaying hybrid repertoires of virulence features.

Our results did not show the presence of enterotoxigenic *E. coli* (ETEC)/STEC hybrid pathotypes among the strains investigated, which were instead previously identified in the bordering area [12]. On the other hand, our results highlight the circulation of certain STEC strains showing the microcin-encoding genes (*mchB*, *mchC*, and *mchF*) and determinants associated with iron metabolism (*ireA* and *lpfA*), as well as *papC*, *senB*, *vat*, and *kpsMII* described in uropathogenic *E. coli* (UPEC). Overall, these findings suggest a role for red deer in the dissemination of hybrid STEC–extraintestinal pathogenic *E. coli* strains. Some of the virulence genes detected through WGS in the STEC strains from red deer have been described as located on mobile genetic elements, particularly on pathogenicity islands (*lpfA* and *ireA*, among others) or plasmids (*senB*), which may have been acquired through horizontal gene transfer and may lead to their further dissemination to other bacteria.

Moreover, in our study, we detected one STEC isolate producing *stx2a* and belonging to O26:H11, a serotype commonly isolated from human disease, including HUS cases [2,4,47].

These findings highlight the need to understand if wild ruminants represent a potential risk to human health, considering that most hunted and culled animals are processed for human consumption. According to the culling plan implemented in the bordering area of SNP, approximately 37.3 tons of deer meat were introduced into the food chain between 2011 and 2016 [49]. Multiple opportunities for *E. coli* contamination occur throughout this peculiar food chain [50]. Indeed, pathogenic bacteria colonize the intestinal tract of the animal or the hide/skin, and contamination from these sources can transfer to the venison product through unhygienic handling of the carcass [51]. Moreover, the meat can become contaminated due to the leakage of the intestinal content following the shooting. The risk of transmission of the infection to humans eventually may occur through the handling of carcasses and raw meat or the consumption of raw or undercooked meat. Our results highlight the need for observance of good hygiene practice while preparing game meat [13,14,50].

The comparison of STEC strains with those circulating in humans in the same area is pivotal for identifying sources of infections and defining the zoonotic transmission. This is made possible by the use of the IRIDA-ARIES national platform [26], which comprises sequencing data from STEC isolated from multiple sources in Italy, allowing the comparison of STEC isolates ‘sequences in a One Health manner. The analysis of the human STEC sequencing data present as of today in the platform revealed the presence of strains with similar characteristics to those isolated from red deer. In particular, several O26:H11 STEC strains have been isolated in Lombardy from human cases, sequenced and included in the IRIDA-ARIES platform, mostly possessing the same *stx2a* as the strain from red deer described in this study. This is not surprising, as STEC O26 is very common in human cases of disease. However, no cgMLST clusters have been identified so far among STEC strains from humans and from red deer described here. Nonetheless the IRIDA-ARIES system enables the continuation of the One Health surveillance, to understand the zoonotic potential of these strains.

We acknowledge that our study had several limitations. The methodology used in this study, including freezing at −20 °C and storing fecal matter for one month, may have limited the viability of STEC strains and further studies based on viability of STEC strains in fecal samples according to time of storage may be suggested to confirm if underestimation of the actual STEC prevalence may have occurred. The bacteriological reference method used is qualitative and does not foresee the quantification of STEC load in fecal samples. Future studies focusing on STEC quantification may help clarify the risk of fecal dissemination and the transmission by red deer. In this study, when several colonies from a specific sample were obtained, a single colony was further submitted to WGS, since they all possessed the same *stx* gene profile. Considering that red deer, like cattle, may carry multiple STEC strains simultaneously [52], the single-colony approach with only one STEC colony per positive fecal sample subjected to WGS may have underestimated within-host strain diversity of the STEC strains circulating in the study population. Future studies applying metagenomic sequencing or sequencing multiple colonies per animal would help to capture a more comprehensive picture of STEC heterogeneity in wildlife populations.

The animals tested were all hunted during a single autumn and winter hunting season. Therefore, the seasonal dynamics of STEC shedding and persistence in the red deer population as previously reported for cattle [53] were not evaluated. Given that STEC shedding is known to be influenced by temperature, diet, and stress, the lack of longitudinal data has restricted the ecological interpretation.

Lastly, it would be interesting to carry out a comparative phylogenomic study including the STEC strains isolated in this study, and livestock and human STEC strains to better understand potential for cross-species transmission, especially since many STEC serotypes circulate widely in both wildlife and livestock.

## 5. Conclusions

Overall, our findings highlight the need for a One Health approach focusing on the understanding of the zoonotic potential of STEC from wild ruminants, comparing data from local hospitals, farms, and water contamination to define transmission risks to humans, domestic animals, and environmental contamination.

## Figures and Tables

**Table 1:** Characteristics of the 92 red deer assayed for the presence of STEC in this study.

Category	Sub-Category	No (%)	STEC Positive No (%) *
Sex	Female	46 (50)	7 (15)
	Male	46 (50)	4 (9)
Age category	Calves	23 (25)	4 (17)
	Yearling	17 (18)	0 (0)
	Adult	52 (57)	7 (13)
Month of sampling	September	11 (12)	1 (9)
	October	23 (25)	2 (9)
	November	30 (33)	3 (10)
	December	28 (30)	5 (18)

* No statistical significance was observed among STEC positivity in the subcategories.

**Table 2:** Characteristics of the 11 STEC strains from red deer determined by WGS analysis.

Strain Code	Sample ID	Serotype	Sequence Type	*stx* Genes Profile	*stx* Subtype	Other Virulence Factors
ED1682	129_RNo*_213	O21:H21	ST56	*stx1*	*stx1c*	*cia*, *ehxA*, *espI*, *gad*, *iha*, *ireA*, *lpfA*, *mchB*, *mchC*, *mchF*, *subAB*, *terC*, *tia*, *traT*
ED1675	46_RNo*_59	O26:H11	ST29	*stx2*	*stx2a*	*astA*, *cba*, *cif*, *cma*, *eae*, *efa1*, *ehxA*, *espA*, *espB*, *espF*, *espJ*, *etpD*, *fyuA*, *gad*, *iha*, *iss*, *iucC*, *iutA*, *lpfA*, *nleA*, *nleB*, *tccP*, *terC*, *tir*, *traT*
ED1680	69_RNo*_261	O27:H30	ST753	*stx2*	*stx2b*	*air*, *cba*, *chuA*, *eilA*, *espI*, *gad*, *hra*, *iha*, *ireA*, *iss*, *mchB*, *mchC*, *mchF*, *subAB*, *terC*, *tia*, *traT*
ED1683	102_RNo*_313	O27:H30	ST753	*stx2*	*stx2b*	*air*, *chuA*, *eilA*, *espI*, *gad*, *iha*, *ireA*, *iss*, *mchB*, *mchC*, *mchF*, *subAB*, *terC*, *tia*, *traT*
ED1685	111_RNo*_726	O27:H30	ST753	*stx2*	*stx2b*	*air*, *cba*, *chuA*, *eilA*, *espI*, *gad*, *iha*, *ireA*, *iss*, *mchB*, *mchC*, *mchF*, *subAB*, *terC*, *tia*, *traT*
ED1667	151_RNo*_117	O54:H45	ST491	*stx2*	*stx2b*	*astA*, *cba*, *celB*, *chuA*, *espI*, *gad*, *ireA*, *iss*, *mchB*, *mchC*, *mchF*, *ompT*, *papC*, *pic*, *senB*, *sitA*, *subAB*, *terC*, *tia*, *traT*, *vat*, *yfcV*
ED1678	104_RNo*_11	O91:H14	ST33	*stx1 stx2*	*stx1a stx2b*	*cba*, *cea*, *ehxA*, *espI*, *espP*, *gad*, *iha*, *ireA*, *iss*, *kpsE*, *kpsMII*, *lpfA*, *saa*, *subAB*, *terC*, *tia*, *traT*
ED1679	77_RNo*_48	O104:H7	ST10075	*stx1*	*stx1c*	*aaiC*, *celB*, *gad*, *lpfA*, *mcbA*, *neuC*, *terC*, *traT*
ED1684	16_RNo*_314	O117:H4	ST56	*stx2*	*stx2b*	*astA*, *cba*, *cia*, *cma*, *cvaC*, *ehxA*, *espI*, *fyuA*, *gad*, *ireA*, *iss*, *lpfA*, *mchB*, *mchC*, *mchF*, *ompT*, *subAB*, *terC*, *tia*, *traT*
ED1666	178_RNo*_94	O146:H28	ST738	*stx2*	*stx2b*	*astA*, *chuA*, *espI*, *gad*, *hra*, *iha*, *ireA*, *lpfA*, *mchB*, *mchC*, *mchF*, *ompT*, *subAB*, *terC*, *traT*, *usp*
ED1676	48_RNo*_191	O?:H8	ST26	*stx2*	*stx2b*	*astA*, *cba*, *cia*, *cma*, *ehxA*, *eilA*, *espI*, *gad*, *iha*, *ireA*, *iss*, *lpfA*, *mchB*, *mchC*, *mchF*, *subAB*, *terC*, *tia*, *traT*

* RNo = registration number; O? = not typeable serogroup.

**Table 3:** Distribution of the 11 STEC serotypes and sequence types according to sex, age, and month of sampling detected in this study.

Category	Sub-Category	O21:H21 (ST56) (*n* = 1)	O26:H11 (ST29) (*n* = 1)	O27:H30 (ST753) (*n* = 3)	O54:H5 (ST491) (*n* = 1)	O91:H14 (ST33) (*n* = 1)	O104:H7 (ST10075) (*n* = 1)	O117:H4 (ST nd) (*n* = 1)	O146:H28 (ST738) (*n* = 1)	ONT:H8 (ST26) (*n* = 1)
Sex	Female	1		2	1	1		1	1	
	Male		1	1			1			1
Age category	Calves			1	1			1	1	
	Yearling									
	Adult	1	1	2		1	1			1
Month of sampling	September							1		
	October		1							1
	November			1			1		1	
	December	1		2	1	1				

## Data Availability

The original contributions presented in this study are included in the article. Further inquiries can be directed to the corresponding author.

## Abbreviations

The following abbreviations are used in this manuscript:

AD	allelic distances
BPW	buffered peptone water
CAC1	Alpine Hunting District of Ponte di Legno
cgMLST	core genome multilocus sequence typing
EAEC	enteroaggregative *E. coli*
EIEC	enteroinvasive *E. coli*
ETEC	enterotoxigenic *E. coli*
HUS	hemolytic uremic syndrome
MLST	multilocus sequence typing
SNP	Stelvio National park
ST	sequence type
STEC	Shiga toxin-producing *Escherichia coli*
UPEC	uropathogenic *E. coli*
WGS	Whole-genome sequencing
